# Airspaces-derived exosomes contain disease-relevant protein signatures in a mouse model of cystic fibrosis (CF)-like mucoinflammatory lung disease

**DOI:** 10.3389/fphar.2024.1460692

**Published:** 2024-09-25

**Authors:** Yun Mao, Amol Suryawanshi, Sonika Patial, Yogesh Saini

**Affiliations:** ^1^ Department of Population Health and Pathobiology, College of Veterinary Medicine, North Carolina State University, Raleigh, NC, United States; ^2^ Comparative and Molecular Pathogenesis Branch, Division of Translational Toxicology, National Institute of Environmental Health Sciences, Research Triangle Park, Durham, NC, United States

**Keywords:** exosome, proteomics, airspace, *Scnn1b*-Tg+, lung, mucoinflammation

## Abstract

Exosomes, membrane-bound extracellular vesicles, ranging from approximately 30–200 nm in diameter, are released by almost all cell types and play critical roles in intercellular communication. In response to the prevailing stress, the exosome-bound protein signatures vary in abundance and composition. To identify the bronchoalveolar lavage fluid (BALF) exosome-bound proteins associated with mucoinflammatory lung disease and to gain insights into their functional implications, we compared BALF exosomes-derived proteins from adult *Scnn1b* transgenic (*Scnn1b*-Tg+) and wild type (WT) mice. A total of 3,144 and 3,119 proteins were identified in BALF exosomes from *Scnn1b*-Tg+ and WT mice, respectively. Using cutoff criteria (Log_2_ fold-change > 1 and adjusted *p*-value < 0.05), the comparison of identified proteins revealed 127 and 30 proteins that were significantly upregulated and downregulated, respectively, in *Scnn1b*-Tg+ versus WT mice. In addition, 52 and 27 proteins were exclusively enriched in *Scnn1b*-Tg+ and WT mice, respectively. The identified exosome-bound proteins from the homeostatic airspaces of WT mice were mostly relevant to the normal physiological processes. The protein signatures enriched in the BALF exosomes of *Scnn1b*-Tg+ mice were relevant to macrophage activation and mucoinflammatory processes. Ingenuity pathway analyses revealed that the enriched proteins in *Scnn1b*-Tg+ mice contributed to the inflammatory responses and antimicrobial defense pathways. Selective proteins including, RETNLA/FIZZ1, LGALS3/Galectin-3, S100A8/MRP8, and CHIL3/YM1 were immunolocalized to specific cell types. The comparative analysis between enriched BALF exosome proteins and previously identified differentially upregulated genes in *Scnn1b*-Tg+ versus WT mice suggested that the compartment-/cell-specific upregulation in gene expression dictates the enrichment of their respective proteins in the lung airspaces. Taken together, this study demonstrates that the BALF exosome-bound protein signatures reflect disease-relevant disturbances. Our findings suggest that the exosomes carry disease-relevant protein signatures that can be used as a diagnostic as well as predictive biomarkers for mucoinflammatory lung disease.

## 1 Introduction

Mucoinflammatory lung diseases including, cystic fibrosis (CF), chronic obstructive pulmonary disease (COPD), primary ciliary dyskinesia (PCD), and non-cystic fibrosis bronchiectasis, are characterized by mucus plugging, impaired mucociliary clearance (MCC), airway inflammation, and recurrent infections (Mall et al., 2018; Boucher, 2019; Burgel et al., 2009). Most of these pathological features of mucoinflammatory lung diseases are displayed by *Scnn1b* transgenic (*Scnn1b*-Tg+) mice (Mall et al., 2004; Mall et al., 2008). The *Scnn1b*-Tg+ mouse overexpresses *sodium channel non-voltage-gated 1, beta subunit* (*Scnn1b*) transgene in club cell secretory protein (CCSP) expressing airway epithelial cells. This overexpression of *Scnn1b* transgene causes hyperabsorption of sodium ions and, as a consequence, establishes an osmotic gradient that draws water into the airway epithelial cells leading to airway surface liquid dehydration, mucin hyperconcentration, and impaired MCC (Mall et al., 2004; Mall et al., 2008). In our previous report, transcriptomic analyses on whole lung and alveolar macrophages revealed differential gene signatures in the *Scnn1b*-Tg+ versus wild-type (WT) mice (Saini et al., 2014). However, apart from selective cytokine analyses (Mall et al., 2004; Mall et al., 2008; Lewis et al., 2020a; Choudhary et al., 2021a; Saini et al., 2016; Mao et al., 2023; Saini et al., 2018; Lewis et al., 2020b; Lewis et al., 2017), comprehensive analyses of airspace protein contents between *Scnn1b*-Tg+ versus WT mice have not yet been conducted.

Exosomes are nano-sized extracellular vesicles (30–200 nm) released by almost all cell types and play critical roles in intercellular communications (Gurung et al., 2021; McVey et al., 2019; Théry et al., 2002). Exosomes are known to regulate homeostatic as well as pathological processes in various organ systems (Théry et al., 2002; Kalluri and LeBleu, 2020; Pathan et al., 2018; Holtzman and Lee, 2020). Multi-omics approaches including transcriptomics, metabolomics, and proteomics, have significantly enhanced our understanding of the exosome contents and their biological relevance during pathophysiological processes (Shaba et al., 2022; Chitoiu et al., 2020). Increasing evidence suggests that bronchoalveolar lavage fluid (BALF) exosomes contribute to inflammatory outcomes in various lung diseases, including asthma, COPD, CF, and PCD (Torregrosa Paredes et al., 2012; Rollet-Cohen et al., 2018; Kaur et al., 2021). Additionally, BALF-derived exosomal miRNAs from COPD patients showed unique miRNA signatures as compared to the healthy subjects, indicating their potential use as biomarkers to track the disease progression (Kaur et al., 2021). Recent studies indicate that exosomal proteomic analyses can be used to gain insights into lung diseases (Rollet-Cohen et al., 2018; Choudhary et al., 2021b; Velázquez-Enríquez et al., 2021). Our recent study revealed that exosome-bound protein signatures from ozone-stressed airspaces reflect the molecular processes involved in mucoinflammatory lung disease (Choudhary et al., 2021b).

In this study, to gain insights into the proteomic differences and their association with the pathological endpoints, we compared exosome-bound protein signatures between *Scnn1b*-Tg+ and WT adult mice. We hypothesized that alterations in the composition of exosome-bound protein signatures in *Scnn1b*-Tg+ mice are suggestive of mucoinflammatory lung disease. To test this hypothesis, exosomes harvested from the BALF of adult WT and *Scnn1b*-Tg+ mice were subjected to liquid chromatography-mass spectrometry (LC-MS/MS) followed by bioinformatic analyses. The results from this study provide critical insights into the relevance of exosome-bound protein signatures in the pathological manifestation of mucoinflammatory lung disease in this transgenic mouse model and suggest their potential application as diagnostic and predictive biomarkers for mucoinflammatory diseases.

## 2 Materials and methods

### 2.1 Animal husbandry

Male *Scnn1b*-Tg+ mice strain was procured from the Jackson Laboratory [Stock Number, 006438; Strain name, *B6.Cg-Tg(Scgb1a1-Scnn1b)6608Bouc/J*, Bar Harbor, ME]. All mice used in this study have a background of C57BL/6. The age of the mice were 6–7 weeks old. Mice were housed in individually ventilated, hot-washed cages on a 12-h light/12-h dark cycle at the Laboratory Animal Resources (LAR) facility at the College of Veterinary Medicine at North Carolina State University (NCSU). Animals were provided food and water *ad libitum*. All animal experiments were performed per NCSU Institutional Animal Care and Use Committee (IACUC) approval.

### 2.2 Exosome collection from bronchoalveolar lavage fluid (BALF)

**FIGURE 1:**
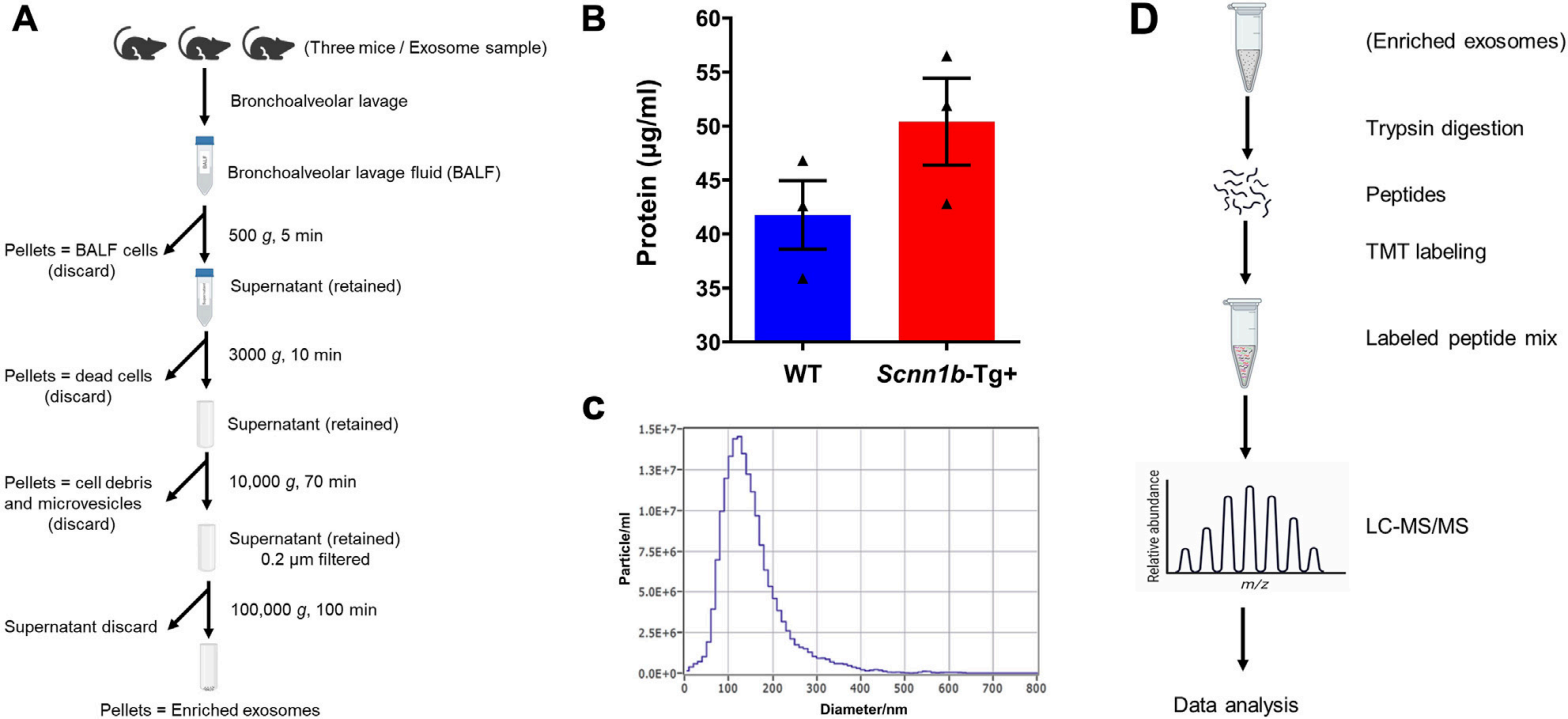
Outline of exosome isolation, characterization, and analysis approach to elucidate exosomal protein enrichment. **(A)** Schematic diagram of exosome isolation using differential ultracentrifugation. The diagram was created via www.biorender.com. **(B)** Total protein contents (μg/ml) in the BALF exosomes harvested from *Scnn1b*-Tg+ and WT mice (N = 3 for each genotype). Statistical analysis was performed by unpaired *t*-test. **(C)** The representative particle size distribution of exosomes was evaluated by a Nanoparticle tracking analyzer. The exosome samples collected in this study had an average diameter of 160.30 ± 4.85 nm. Error bars represent Mean ± Standard Error of the Mean (SEM). N = 3 for each genotype. **(D)** Flow diagram involved in exosome processing for proteomics data analyses. The diagram was created via www.biorender.com.

BALF exosomes were collected by differential ultracentrifugation following a previously reported procedure (Choudhary et al., 2021b). Each exosome sample represents exosomes collected from BALF harvested from a total of three adult male mice. All the centrifugation steps were carried out at 4°C to prevent protein degradation (Figure 1A). The particle size distribution of exosomes was evaluated by a Nanoparticle tracking analyzer (ZetaView-version 8.05.16 SP3 software, Particle Metrix GmbH, Germany).

### 2.3 Sample preparation and data acquisition

Proteomic and data analyses were carried out by mass spectrometry (MS)-based quantitative proteomics as described previously (Choudhary et al., 2021b). Briefly, total protein from each sample was subjected to reduction, alkylation, and digestion using single-pot, solid-phase-enhanced sample preparation (SP3) (Hughes et al., 2019) with sequencing grade modified porcine trypsin (Promega, Madison, WI). Tryptic peptides were subsequently separated on an in-line 150 × 0.075 mm column filled with reverse phase XSelect CSH C18 2.5 µm resin (Waters Corporation, Milford, MA) using a Thermo UltiMate 3,000 RSLCnano system (Thermo Fisher Scientific, Waltham, MA). Peptides were eluted using a 60 min gradient from 98:2 to 65:35 buffer A (0.1% formic acid, 0.5% acetonitrile): B (0.1% formic acid, 99.9% acetonitrile) ratio. Eluted peptides were ionized by electrospray at 2.2 kV and subsequently analyzed using an Orbitrap Exploris 480 mass spectrometer (Thermo Fisher Scientific, Waltham, MA). To construct a chromatogram library (Searle et al., 2018), we collected six gas-phase fractions on the Orbitrap Exploris using 4 m/z data-independent acquisition (DIA) spectra. Each spectrum employed the 4 m/z precursor isolation windows at 30,000 resolutions (normalized AGC target of 100% and maximum inject time of 66 m). After each DIA duty cycle, precursor spectra were obtained across the entire m/z range of the gas-phase fraction. For wide-window acquisitions, Orbitrap Exploris was set up to capture a precursor scan followed by 50 × 12 m/z DIA spectra.

### 2.4 Data analysis

Proteins were searched against the UniProt *Mus musculus* database (February 2024) using Spectronaut (Biognosys version 18.6) and employing the directDIA method. The data was acquired with quantity at the MS2 level, cross-run normalization to false, and protein grouping quantification based on median peptide and precursor abundance. Protein MS2 intensity values were evaluated for quality using ProteiNorm (Graw et al., 2020). The data was normalized using Cyclic Loess (Ritchie et al., 2015) and subjected to analysis using proteoDA (Thurman et al., 2023). Statistical analysis was performed by applying Linear Models for Microarray Data (limma) with empirical Bayes (eBayes) smoothing to the standard errors (Ritchie et al., 2015). Proteins meeting the criteria of an adjusted *p*-value < 0.05 and a Log_2_FC > 1 were considered statistically significant.

### 2.5 Biological pathway and protein interaction network analyses

Canonical pathways and biological networks were analyzed by Ingenuity Pathway Analysis (IPA) software (Qiagen, Redwood City, CA). The cutoff criteria (Log_2_FC > 1; adjusted *p*-value < 0.05) was used to screen proteins for analyses. Accordingly, differentially enriched proteins were uploaded into IPA software to analyze the enrichment of canonical pathways. The proteins with high abundance and low abundance were uploaded into IPA software to analyze their contribution to the enrichment of diseases and functions. The protein-protein interaction (PPI) network was analyzed by STRING (https://string-db.org), which is a database containing previously known and predicted interactions between proteins based on the provided input information (Szklarczyk et al., 2019). We used it to construct the PPI networks for differently enriched exosomal proteins with an interaction score greater than 0.4 or 0.7 as the cutoff value. The Cytoscape is a software for the network visualization (Kohl et al., 2011). Hub proteins in the network were identified by using the CytoHubba plugin of Cytoscape (Chin et al., 2014). Node degrees were ranked by score value.

### 2.6 Immunohistochemical localization of selected exosomal proteins

Left lung lobes were harvested from a separate cohort of mice and fixed in 10% neutral-buffered formalin. The formalin-fixed lung lobes were paraffin-embedded, cut into 5-μm sections, and placed on glass slides. Sections were subjected to immunohistochemical staining, as previously described (Choudhary et al., 2021b; Choudhary et al., 2021c; Lewis et al., 2020c). Rabbit polyclonal resistin-like alpha (RETNLA/FIZZ1) primary antibody (ab39626; 1:1,000 dilution; Abcam, Cambridge, MA), rabbit monoclonal S100 calcium-binding protein A8 (S100A8/MRP8) primary antibody (ab92331; 1:1,000 dilution; Abcam), rabbit monoclonal chitinase-3-like protein 3 (CHIL3/YM1) primary antibody (ab230610; 1:50,000 dilution; Abcam), and rabbit monoclonal galectin-3 [lectin, galactoside-binding, soluble, 3 (LGALS3)] primary antibody (ab76245; 1:1,000 dilution; Abcam) were used to assess the immunohistochemical localization of the corresponding antigens, respectively. The stained proteins were visualized using the ImmPACT NovaRED Substrate Kit (SK-4805; Vector Laboratories, Burlingame, CA). The immunostained slides were examined and photographed under the 4Χ or 40Χ objective of a light microscope with a DS-L3 digital camera (Eclipse Ci-L, Nikon Corporation, Tokyo, Japan).

### 2.7 Statistical analyses

Statistical analyses were performed by unpaired *t*-tests between groups. All data were presented as Mean ± Standard Error of the Mean (SEM). A *p*-value of less than 0.05 was considered statistically significant.

## 3 Results

### 3.1 Identification of exosome-bound proteins enriched in *Scnn1b*-Tg+ airspaces

**FIGURE 2:**
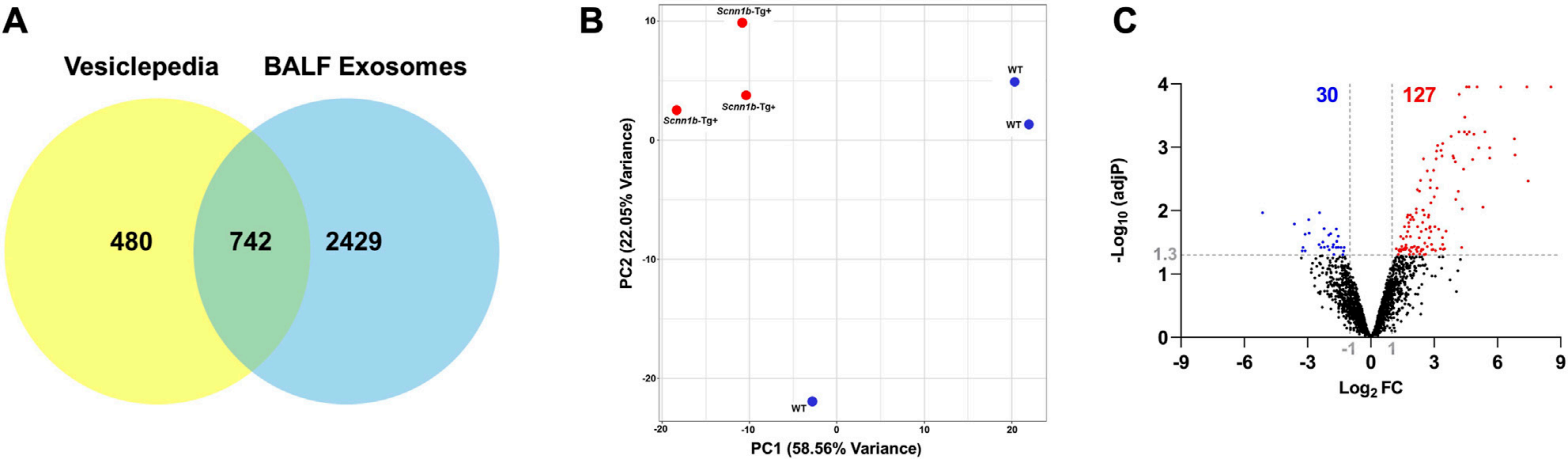
Differential enrichment of BALF exosome-bound proteins in *Scnn1b*-Tg+ versus WT mice. **(A)** Venn diagram depicts that 742 out of 1,222 exosome-specific markers obtained from the Vesiclepedia database were identified in exosomes harvested in this study. **(B)** Two-dimensional principal component (PC) analysis plot using PC1 and PC2 on differentially enriched proteins (after normalization) in exosomes from the *Scnn1b*-Tg+ and WT mice. **(C)** Volcano plots representing differentially abundant proteins in *Scnn1b*-Tg+ versus WT mice (Log_2_FC > 1; adjusted *p*-value < 0.05).

Exosomes were isolated from cell-free BALF using a differential centrifugation approach (Figure 1A). As shown in Figure 1B, the total protein concentrations in the exosomes from *Scnn1b*-Tg+ male mice (50.40 ± 4.03 μg/ml) trended higher as compared to WT male mice (41.77 ± 3.17 μg/ml), but the differences were not statistically significant. The average exosome size and concentration from male mice were 160.30 ± 4.85 nm and 4.0 × 10^10^ particle/ml, respectively (Figure 1C; Supplementary Figure S1). The differences of size and concentration were not statistically significant between *Scnn1b*-Tg+ and WT male mice (Supplementary Figure S1). The isolated exosomes were subjected to proteomics data generation and bioinformatic analysis (Figure 1D). Next, we retrieved a list of 1,222 exosome-specific protein signatures from the Exocarta Vesiclepedia database (Keerthikumar et al., 2016; Simpson et al., 2012). The comparative analysis between the Exocarta Vesiclepedia database and identified proteins from this study revealed that 742 out of 1,222 exosome-specific protein signatures were detected in BALF exosomes obtained in this study (Figure 2A; Supplementary Table S1). Out of the 742 exosome-specific protein signatures, 732 protein signatures were detected in BALF from both *Scnn1b*-Tg+ and WT mice, and 10 protein signatures were exclusively enriched in BALF from either *Scnn1b*-Tg+ or WT mice (Supplementary Table S1). Collectively, our data indicate that the exosome-specific protein signatures differ between *Scnn1b*-Tg+ and WT mice BALF, indicating the potential for the identification of novel exosome-based biomarkers for mucoinflammatory diseases.

### 3.2 Differential enrichment of BALF exosome proteins in *Scnn1b*-Tg+ versus WT mice

The top two principal components (PC1 and PC2), which contribute to ∼80% variance, revealed that genotypic difference is the primary driver of differential protein enrichment in the *Scnn1b*-Tg+ versus WT mice (Figure 2B). Next, using cutoff criteria, i.e., Log_2_FC > 1 and adjusted *p*-value < 0.05, we identified differentially enriched protein signatures in BALF exosomes harvested from *Scnn1b*-Tg+ and WT mice. A total of 127 and 30 proteins were upregulated and downregulated, respectively, in *Scnn1b*-Tg+ versus WT mice (Figure 2C). The protein signatures enriched in *Scnn1b*-Tg+ mice BALF are listed in Table 1 (top 50 proteins) and Supplementary Table S2 (entire list). A list of 30 proteins with low abundance in exosomes from *Scnn1b*-Tg+ mice BALF is included in Table 2. Additionally, 52 and 27 proteins were exclusively expressed, respectively, in BALF from *Scnn1b*-Tg+ and WT mice (Supplementary Table S3).

**TABLE 1:** Top 50 protein signatures enriched in exosomes from *Scnn1b*-Tg+ vs WT mice.

S. No	Protein	Description	*Scnn1b*-Tg+ vs WT	
			Log_2_FC	Adjusted *p*-value
1	CD177	CD177 antigen	8.535333081	0.000111525
2	RETNLA	Resistin-like alpha	7.452739491	0.003406803
3	S100A9	Protein S100-A9	7.396934371	0.000111525
4	GP2	Pancreatic secretory granule membrane major glycoprotein GP2	6.83634736	0.001326941
5	CAMP	Cathelicidin antimicrobial peptide	6.807619403	0.000736474
6	OVOS	Ovostatin homolog	6.151830996	0.000111525
7	CLCA1	Calcium-activated chloride channel regulator 1	5.634310913	0.001021086
8	SYT10	Synaptotagmin-10	5.632701688	0.001483315
9	H2AZ1	Histone H2A.Z	5.40131305	0.000572166
10	S100A8	Protein S100-A8	5.313632236	0.008838892
11	CLCA4A	Calcium-activated chloride channel regulator 4A	5.110325176	0.001021086
12	IFITM1	Interferon-induced transmembrane protein 1	5.039991463	0.000111525
13	P01878	Ig alpha chain C region	4.889294718	0.000622468
14	ITGAM	Integrin alpha-M	4.827787114	0.001552748
15	MPO	Myeloperoxidase	4.669957192	0.000572166
16	H4C1	Histone H4	4.659485721	0.000111525
17	CDHR3	Cadherin-related family member 3	4.552854699	0.000622468
18	H2AC4	Histone H2A type 1-B	4.535089171	0.000111525
19	IFITM3	Interferon-induced transmembrane protein 3	4.451589158	0.00033463
20	SMIM5	Small integral membrane protein 5	4.434891769	0.0005721
21	SCGB3A1	Secretoglobin family 3A member 1	4.394812224	0.002216533
22	NGP	Neutrophilic granule protein	4.332851101	0.009367
23	SNX27	Sorting nexin-27	4.301703523	0.038308792
24	H3-5	Histone H3.3C	4.185589525	0.00014661
25	GGT1	Glutathione hydrolase 1 proenzyme	4.171607329	0.000572166
26	SCGB3A2	Secretoglobin family 3A member 2	4.149012863	0.004980615
27	FBP1	Fructose-1,6-bisphosphatase 1	4.029126623	0.00663156
28	PGLYRP1	Peptidoglycan recognition protein 1	4.00775214	0.001697105
29	IFITM2	Interferon-induced transmembrane protein 2	3.927146371	0.001483315
30	LTF	Lactotransferrin	3.893074654	0.001372762
31	H2BC7	Histone H2B type 1-F/J/L	3.802902687	0.000672995
32	SDCBP2	Syntenin-2	3.565076588	0.021087872
33	FTL1	Ferritin light chain 1	3.497095261	0.039729557
34	HBB-B2	Hemoglobin subunit beta-2	3.436306954	0.040788975
35	Q3UST5	UPF0764 protein C16orf89 homolog	3.409648338	0.034030527
36	PROM1	Prominin-1	3.402043453	0.000871891
37	BPGM	Bisphosphoglycerate mutase	3.392276358	0.026596831
38	H1-3	Histone H1.3	3.374535178	0.001372762
39	BPIFB1	BPI fold-containing family B member 1	3.32781727	0.001116953
40	H1-4	Histone H1.4	3.297469284	0.039788241
41	LIPA	Lysosomal acid lipase/cholesteryl ester hydrolase	3.216926446	0.019686067
42	SLC13A2	Solute carrier family 13 member 2	3.165505305	0.00093433
43	ADAM8	Disintegrin and metalloproteinase domain-containing protein 8	3.138632543	0.00442994
44	TMC5	Transmembrane channel-like protein 5	3.115777695	0.001151024
45	PIGR	Polymeric immunoglobulin receptor	3.098318421	0.001521762
46	UBA6	Ubiquitin-like modifier-activating enzyme 6	3.093256604	0.001521762
47	SLC26A4	Pendrin	3.060772652	0.042625922
48	CLCA3A1	Calcium-activated chloride channel regulator 3A-1	3.032682023	0.018009253
49	H1-2	Histone H1.2	3.000890316	0.006046636
50	TMC4	Transmembrane channel-like protein 4	2.976401595	0.002315094

**TABLE 2:** The 30 protein signatures enriched in WT vs *Scnn1b*-Tg+ mice.

S. No	Protein	Description	WT vs *Scnn1b*-Tg+	
			Log_2_FC	Adjusted *p* -value
1	HOPX	Homeodomain-only protein	5.139671138	0.01086791
2	COL1A1	Collagen alpha-1(I) chain	3.627324443	0.016273422
3	GPX8	Probable glutathione peroxidase 8	3.254116224	0.042961632
4	REEP3	Receptor expression-enhancing protein 3	3.219550019	0.038308792
5	MDK	Midkine	3.119057892	0.042961632
6	NAE1	NEDD8-activating enzyme E1 regulatory subunit	3.114070818	0.023672771
7	LBR	Delta(14)-sterol reductase LBR	2.942787067	0.01398619
8	SEC63	Translocation protein SEC63 homolog	2.926693045	0.022716438
9	RPS27A	Ubiquitin-ribosomal protein eS31 fusion protein	2.453512424	0.034296028
10	SQLE	Squalene monooxygenase	2.438421957	0.01086791
11	APLP2	Amyloid beta precursor like protein 2	2.362205668	0.038308792
12	SRR	Serine racemase	2.273631542	0.031146328
13	AGPAT1	1-acyl-sn-glycerol-3-phosphate acyltransferase alpha	2.211173923	0.019280599
14	SMPD2	Sphingomyelin phosphodiesterase 2	2.20601157	0.037980522
15	NDUFB10	NADH dehydrogenase [ubiquinone] 1 beta subcomplex subunit 10	2.041640721	0.036428574
16	TMEM123	Porimin	1.996551177	0.024310761
17	TMPO	Lamina-associated polypeptide 2, isoforms beta/delta/epsilon/gamma	1.970496063	0.041532632
18	CYP4V2	Cytochrome P450 4V2	1.900679991	0.023155983
19	UFL1	E3 UFM1-protein ligase 1	1.806070199	0.03768799
20	SSR3	Translocon-associated protein subunit gamma	1.770025725	0.034296028
21	UBTF	Nucleolar transcription factor 1	1.751268947	0.049073478
22	SSBP1	Single-stranded DNA-binding protein, mitochondrial	1.64259622	0.038308792
23	TST	Thiosulfate sulfurtransferase	1.630912728	0.019516204
24	SCRN2	Secernin-2	1.612228832	0.030440706
25	RTKN2	Rhotekin-2	1.547259789	0.025325551
26	ESYT3	Extended synaptotagmin-3	1.546856843	0.037980522
27	SRPRA	Signal recognition particle receptor subunit alpha	1.415971252	0.038308792
28	OAT	Ornithine aminotransferase, mitochondrial	1.323831996	0.049696055
29	FBLN5	Fibulin-5	1.295142118	0.043186521
30	EHD2	EH domain-containing protein 2	1.268122226	0.038308792

### 3.3 Macrophage activation-associated proteins are differently enriched in BALF exosome of *Scnn1b*-Tg+ versus WT mice

**FIGURE 3:**
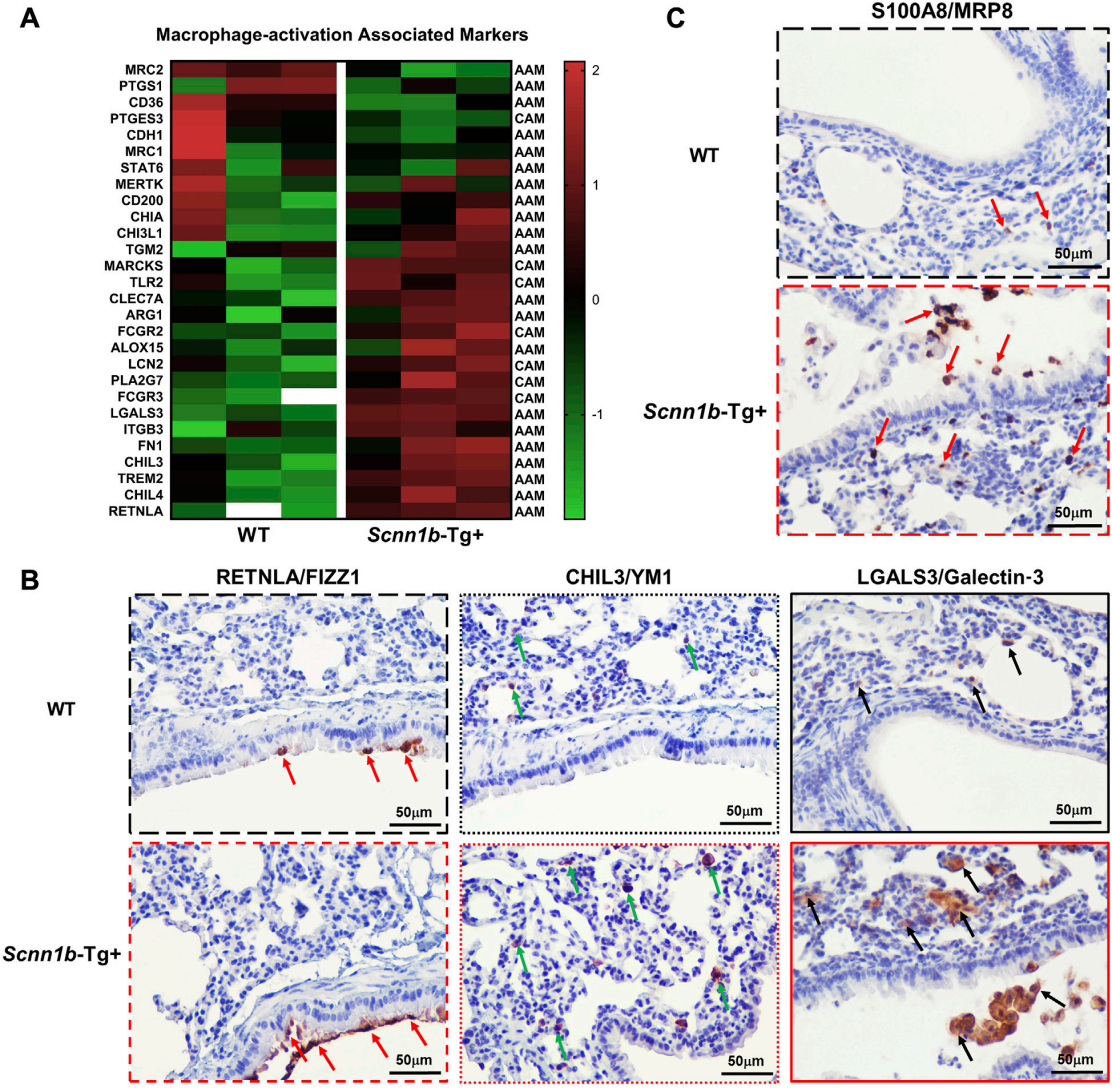
Exosome-bound proteins associated with activated macrophages and neutrophils in *Scnn1b*-Tg+ mice. Heat maps for normalized protein abundance values (Z-scores) of exosome-bound proteins associated with activated macrophages. **(A)** Classically activated macrophages (M1; CAM)- and alternatively activated macrophages (M2; AAM)-specific protein signatures were differently enriched in BALF exosome of *Scnn1b*-Tg+ and WT mice. Higher and lower expressions of each protein are denoted by red and green colors, respectively. The white blanks indicate the levels below the limit of detection. **(B)** Immunohistochemical staining of lung sections for cell-specific expression of AAM activation-associated signatures, FIZZ1 (RETNLA), YM1 (CHIL3), and Galectin-3 (LGALS3). Red arrows depict the FIZZ1-stained epithelial cells. Green arrows depict the YM1 staining in alveolar macrophages. Black arrows depict the Galectin-3-stained macrophages. **(C)** Immunohistochemical staining of lung sections for neutrophil-specific expression of MRP8 (S100A8). Red arrows point to the MRP8-stained neutrophils.

Through a manual literature search, we prepared a list of protein signatures associated with macrophage activation and compared their levels in the exosomes from WT and *Scnn1b*-Tg+ mice BALF. The classically-activated macrophages CAM; M1-associated signatures, i.e., myristoylated alanine-rich C-kinase substrate (MARCKS), toll-like receptor 2 (TLR2), low-affinity immunoglobulin gamma Fc region receptor II (FCGR2), lipocalin 2 (LCN2), platelet-activating factor acetylhydrolase (PLA2G7), and FCGR3, were upregulated in BALF of *Scnn1b*-Tg+ mice as compared to WT mice (Figure 3A). The alternatively-activated macrophages AAM; M2 associated signatures, i.e., RETNLA/FIZZ1, TREM2, CHIL3/YM1, CHIL4, and LGALS3/Galectin-3 were upregulated in BALF from *Scnn1b*-Tg+ mice compared to WT mice (Figure 3A). Macrophage activation protein signatures, i.e., C-type mannose receptor 2 (MRC2) and prostaglandin G/H synthase 1 (PTGS1), were downregulated in BALF from *Scnn1b*-Tg+ mice compared to WT mice (Figure 3A).

Next, to identify the cellular source of RETNLA/FIZZ1, CHIL3/YM1, and Galectin-3/LGALS3 proteins in exosomes, we conducted immunohistochemical staining on lung sections from *Scnn1b*-Tg+ and WT mice. RETNLA/FIZZ1 immunolocalization was restricted to the airway epithelial cells of *Scnn1b*-Tg+ mice (Figure 3B; Supplementary Figure S2). The intensity of CHIL3/YM1 and LGALS3/Galectin-3 were greater in the alveolar macrophages of *Scnn1b*-Tg+ mice versus WT mice (Figure 3B; Supplementary Figure S2). Myeloid-related protein 8 (MRP8/S100A8), is a neutrophil- and monocyte-specific protein (Frosch et al., 2000; Ryckman et al., 2003). To determine the cellular source of MRP8 in airspaces-derived exosomes, we performed immunohistochemical staining on lungs from *Scnn1b*-Tg+ and WT mice (Figure 3C; Supplementary Figure S2). The MRP8-stained cells in *Scnn1b*-Tg+ mice were exclusively neutrophils (Figure 3C; Supplementary Figure S2).

### 3.4 Mucoinflammation and mucous cell metaplasia (MCM) signatures are differently enriched in BALF exosome of *Scnn1b*-Tg+ versus WT mice

**FIGURE 4:**
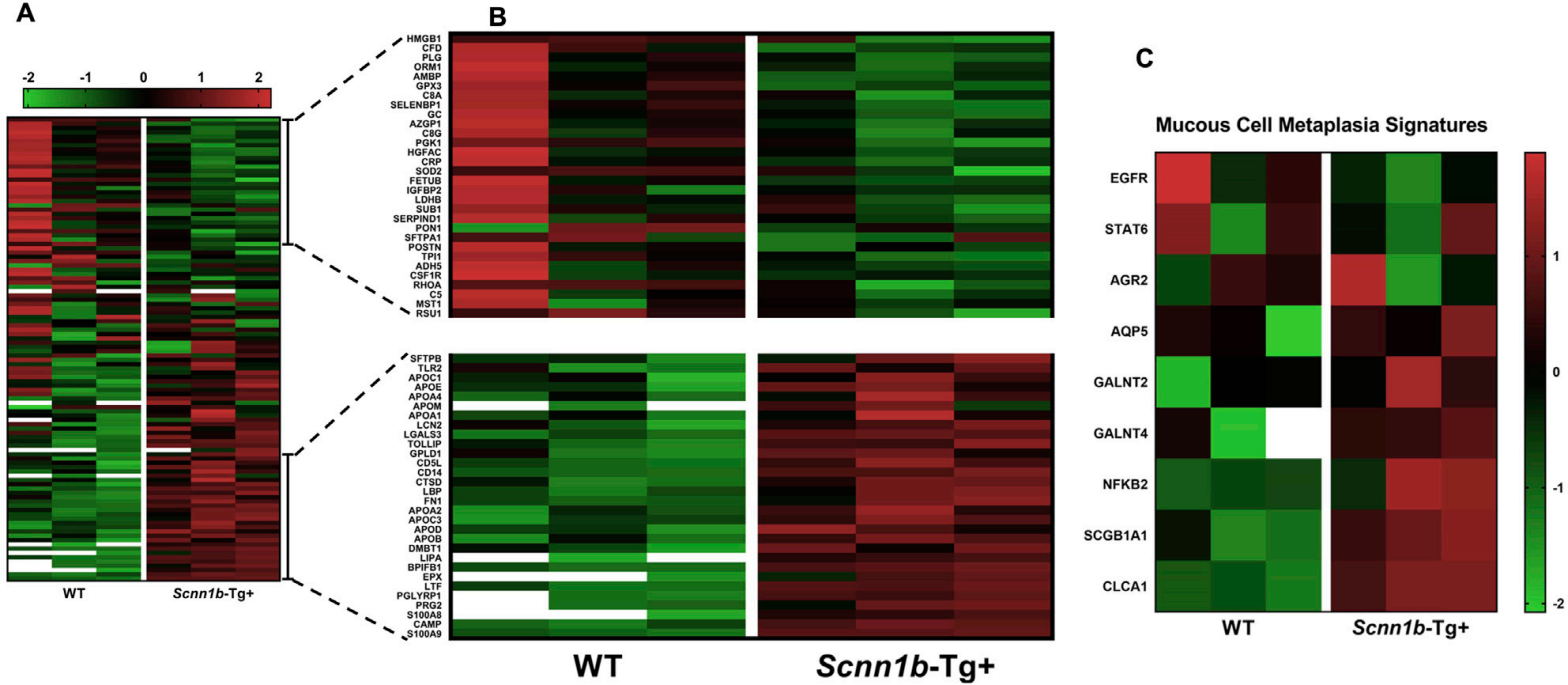
Heat maps for normalized protein abundance values (Z-scores) of exosome-bound proteins relevant to the mucoinflammatory disturbances in mice and humans. **(A)** Low-resolution heat map of exosome-bound protein signatures associated with mucoinflammatory disease in mice and humans (A high-resolution heat map with protein names is provided as Supplementary Figure S3). The heat map representing low abundance [**(B)**; top panel] or enriched [**(B)**; bottom panel] protein signatures in BALF exosomes from *Scnn1b*-Tg+ mice. **(C)** Heat map for exosome-bound protein signatures associated with mucous cell metaplasia. The white blanks indicate the levels below the limit of detection.

We prepared a list of signatures known to be associated with mucoinflammatory lung diseases in mice and humans (Choudhary et al., 2021b; Choudhary et al., 2021d; Saini et al., 2014) and compared their levels in the airspace-derived exosomes from WT and *Scnn1b*-Tg+ mice. Our analyses revealed significant enrichment of the mucoinflammatory signatures, i.e., S100A9, cathelicidin antimicrobial peptide (CAMP), S100A8, peptidoglycan recognition protein 1 (PGLYRP1), lactotransferrin (LTF), BPI fold-containing family B member 1 (BPIFB1), lysosomal acid lipase/cholesteryl ester hydrolase (LIPA), apolipoprotein B-100 (APOB), apolipoprotein C-III (APOC3), cluster of differentiation 14 (CD14), CD5 antigen-like (CD5L), cathepsin D (CTSD), and LGALS3, in BALF exosome of *Scnn1b*-Tg+ versus WT mice (Figures 4A, B; Supplementary Figure S3; Supplementary Table S4). Although non-significantly, some mucoinflammatory protein signatures, i.e., high mobility group protein B1 (HMGB1), complement factor D (CFD), plasminogen, orosomucoid-1 (ORM1), alpha-1-microglobulin/bikunin precursor (AMBP), and glutathione peroxidase 3 (GPX3), were relatively less abundant, in BALF exosome of *Scnn1b*-Tg+ mice versus WT mice (Figures 4A, B; Supplementary Figure S1; Supplementary Table S4). MCM is a consistent feature of *Scnn1b*-Tg+ lung disease (Mall et al., 2004; Mall et al., 2008; Lewis et al., 2020c). The MCM-associated protein signatures including, secretoglobin family 1A member 1 (SCGB1A1) and calcium-activated chloride channel regulator 1 (CLCA1), were significantly enriched in BALF exosome of *Scnn1b*-Tg+ mice as compared to WT mice (Figure 4C).

### 3.5 Pathway analysis on BALF exosome-bound proteins

**FIGURE 5:**
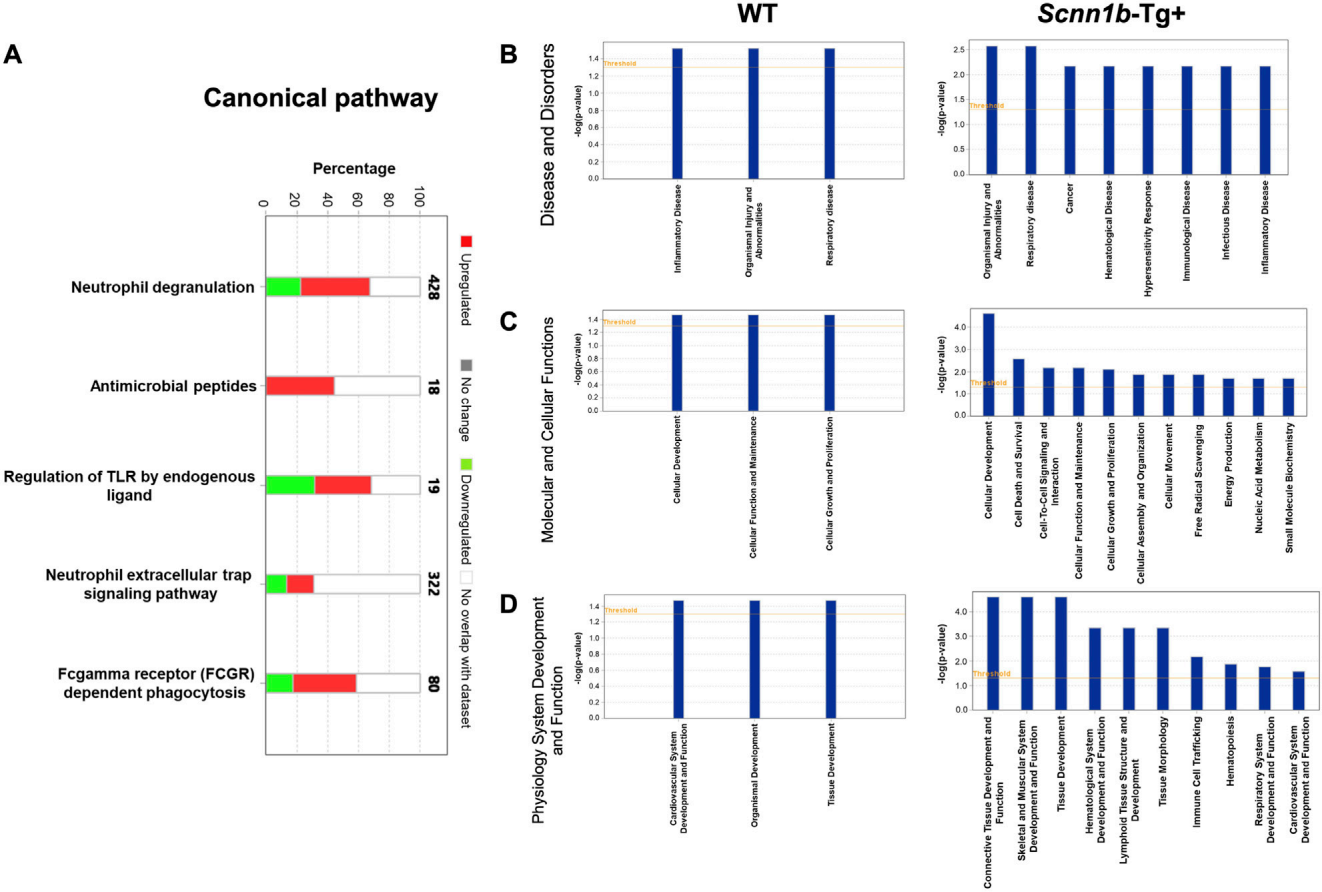
Ingenuity Pathway (IP) analyses of BALF exosome-bound proteins enriched in *Scnn1b*-Tg+ and WT mice. **(A)** IP analysis for the enrichment of canonical pathways influenced by the significantly enriched proteins in *Scnn1b*-Tg+ versus WT mice (Log_2_FC > 1; adjusted *p*-value < 0.05). Stacked bar graph indicates the proportions of total enriched (red) and low abundance (green) proteins associated with a particular pathway. IP analysis for the enrichment of disease and disorders **(B)** Molecular and cellular functions **(C)**, and physiology system development and function **(D)** influenced by the abundant proteins in BALF exosomes from *Scnn1b*-Tg+ and WT mice.

To identify canonical pathways that are enriched due to the abundance of exosome-bound proteins harvested in this study, we employed ingenuity pathways analysis (IPA) approach (cutoff criteria-Log_2_FC > 1 and adjusted *p*-value < 0.05). Our analyses revealed that enriched proteins in BALF exosomes of *Scnn1b*-Tg+ were associated with the upregulation of pathways including neutrophil degranulation, antimicrobial peptides, endogenous ligand-mediated TLR regulation, neutrophil extracellular trap signaling pathway, and Fc-gamma receptor (FCGR) dependent phagocytosis (Figure 5A).

The top diseases and biological functions influenced by the enriched proteins in BALF exosome of *Scnn1b*-Tg+ mice included organismal injury and abnormalities, inflammatory disease, cancer, hematological disease, hypersensitivity response, immunological disease, infectious disease, and respiratory disease (Figure 5B). The top molecular and cellular functions influenced by the enriched proteins in BALF exosome of *Scnn1b*-Tg+ mice included cellular development, cell death and survival, cell-to-cell signaling and interaction, cellular function and maintenance, cellular growth and proliferation, cellular assembly and organization, cellular movement, free radical scavenging, energy production, nucleic acid metabolism, and small molecule biochemistry. In WT mice, the enriched proteins were relevant to cellular development, function, maintenance, growth, and proliferation (Figure 5C). The top physiology systems development and functions associated with the enriched exosomal proteins in *Scnn1b*-Tg+ mice included connective tissue development and function, skeletal and muscular system development and function, tissue development, hematological system development and function, lymphoid tissue structure and development, tissue morphology, immune cell trafficking, hematopoiesis, respiratory system development and function, and cardiovascular system development and function (Figure 5D).

### 3.6 Protein-protein interaction network analysis on BALF exosome-bound proteins

**FIGURE 6:**
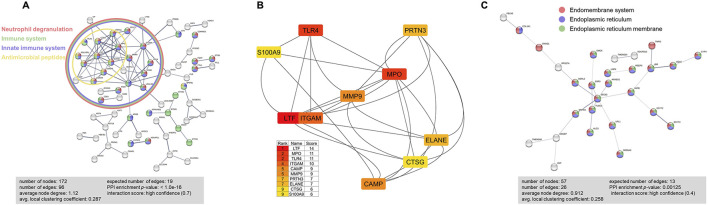
STRING database protein-protein interaction network analyses were performed on the abundant BALF exosome proteins from *Scnn1b*-Tg+ versus WT mice. **(A)** A total of 179 (127 enriched proteins and 52 exclusively abundant in *Scnn1b*-Tg+ mice) protein signatures were used for this analysis. The protein-protein interactions were determined based on evidence, using the high confidence level (0.7) setting. 172 nodes and 96 edges were identified. Disconnected nodes were selected to be hidden. PPI enrichment *p*-value < 1.0e-16. Proteins involved in Neutrophil degranulation (Counts = 36/526; FDR adjusted *p*-value < 1.57e-19), Immune system (Counts = 49/1,615; FDR adjusted *p*-value < 9.34e-14), Innate immune system (Counts = 44/945; FDR adjusted *p*-value < 1.44e-18), and antimicrobial peptides (Counts = 10/84; FDR adjusted *p*-value < 1.41e-06) were enriched. **(B)** Top 10 hub proteins in the network ranked by score value using the Cytoscape-CytoHubba plugin. These proteins included lactotransferrin (LTF), myeloperoxidase (MPO), toll-like receptor 4 (TLR4), integrin subunit alpha M (ITGAM), cathelicidin antimicrobial peptide (CAMP), matrix metalloproteinase 9 (MMP9), proteinase 3 (PRTN3), neutrophil elastase (ELANE), cathepsin G (CTSG), and S100 calcium binding protein A9 (S100A9). **(C)** STRING database protein-protein interaction network analyses on differently expressed proteins that had high abundance and exclusively expressed (total of 57) in WT mice. The protein-protein interactions were determined based on evidence, using the high confidence level (0.4) setting. 57 nodes and 26 edges were identified. Disconnected nodes were selected to be hidden. PPI enrichment *p*-value < 0.00125. Proteins involved in the Endomembrane system (Counts = 32/4360; FDR adjusted *p*-value < 5.15e-07), Endoplasmic reticulum (Counts = 27/1926; FDR adjusted *p*-value < 1.66e-11), and endoplasmic reticulum membrane (Counts = 24/1,100; FDR adjusted *p*-value < 2.67e-13) were enriched.

To identify the protein-protein interaction network, we performed STRING analyses on 179 proteins that were abundant in BALF exosomes from *Scnn1b*-Tg+ mice. These proteins included the 127 significantly enriched proteins in BALF from *Scnn1b*-Tg+ versus WT mice (Log_2_FC > 1; adjusted *p*-value < 0.05), and 52 proteins that were exclusively found in BALF from *Scnn1b*-Tg+ mice. The significantly influenced protein-protein interaction networks due to the enriched protein signatures in the BALF exosomes of *Scnn1b*-Tg+ versus WT mice included neutrophil degranulation, immune system, innate immune system, and antimicrobial peptides (Figure 6A). Furthermore, the top 10 proteins, i.e., LTF, myeloperoxidase (MPO), TLR4, integrin subunit alpha M (ITGAM), CAMP, matrix metalloproteinase 9 (MMP9), proteinase 3 (PRTN3), neutrophil elastase (ELANE), cathepsin G (CTSG), and S100 calcium-binding protein A9 (S100A9), were involved in network ranked by degree method using Cytoscape-CytoHubba plugin (Figure 6B). To further explore the functional relevance of 30 protein signatures, which were more abundant in BALF from WT mice, and 27 protein signatures, which were exclusively present in BALF from WT mice, STRING database protein-protein interaction network analyses were conducted. The output of STRING analysis indicated that the top networks were involved in the endoplasmic reticulum, i.e., endomembrane system, endoplasmic reticulum, and endoplasmic reticulum membrane (Figure 6C).

### 3.7 Comparative analysis between enriched BALF exosome proteins and transcriptomes from *Scnn1b*-Tg+ versus WT mice

**FIGURE 7:**
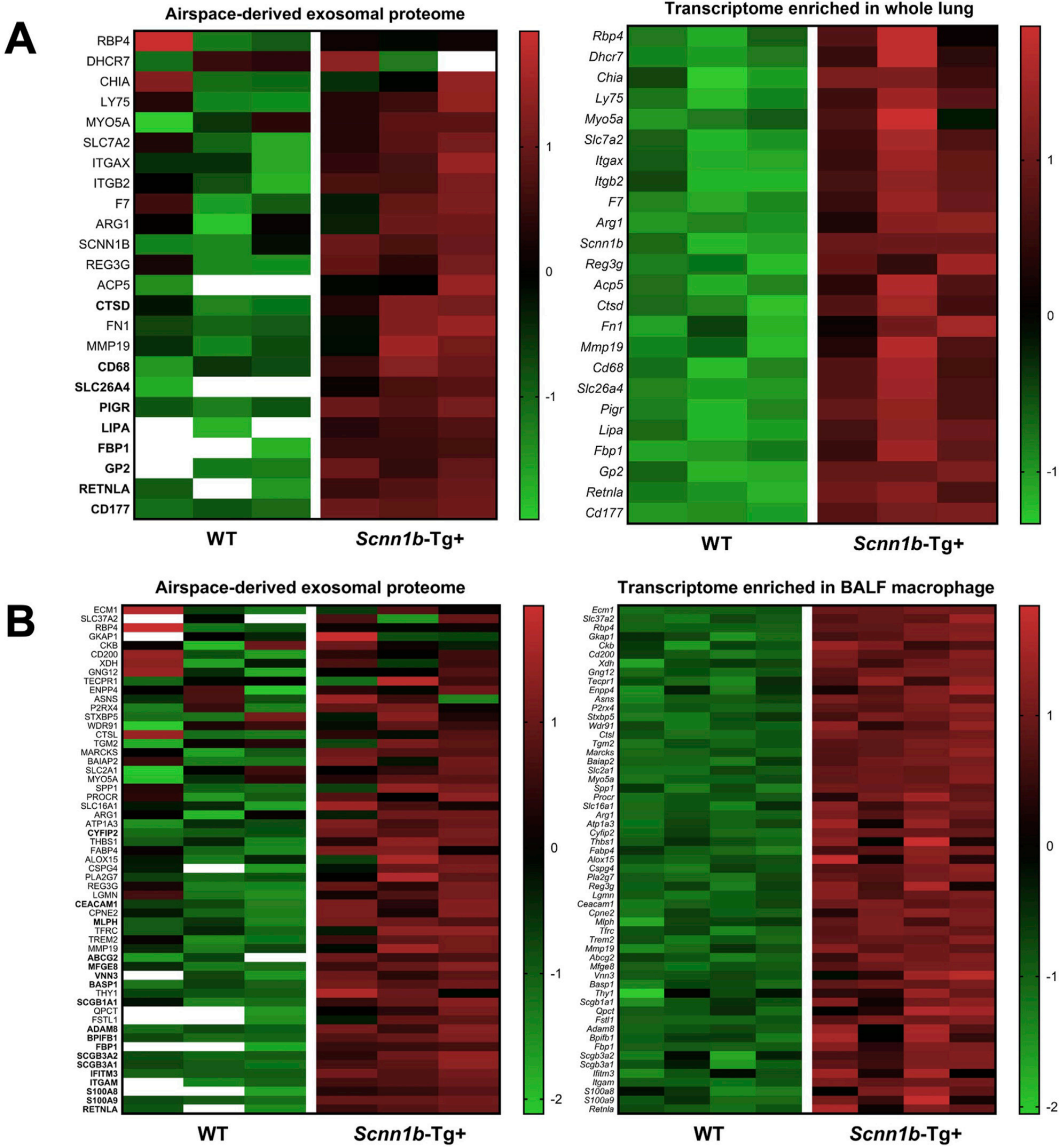
Comparative proteins and transcriptome analysis. Comparative analysis of protein signatures (Log_2_FC > 1 and adjusted *p*-value < 0.05) enriched in *Scnn1b*-Tg+ BALF exosomes and differentially upregulated genes (FC > 2 and adjusted *p*-value < 0.05) in whole lung **(A)** and purified BALF macrophage **(B)** of *Scnn1b*-Tg+ mice. Heat maps for normalized values (Z-scores) of upregulated differentially expressed genes (right panels) from the whole lung and BALF macrophage of *Scnn1b*-Tg+ versus WT mice and normalized values (Z-scores) of corresponding exosome proteins identified in the airspace of *Scnn1b*-Tg+ versus WT mice (left panels). The data with white blank in the heat maps means it is below limit of detection.

To investigate the cellular origin of proteins and ascertain whether the enriched exosomal proteins mirror their upregulation at the mRNA levels, we cross-referenced significantly enriched proteins from *Scnn1b*-Tg+ airspace with the differentially upregulated transcripts from the whole lung and alveolar macrophages (purified BALF macrophages) of *Scnn1b*-Tg+ versus WT mice (Saini et al., 2014). The 127 enriched exosomal proteins (Log_2_FC > 1 and adjusted *p*-value < 0.05) and the 52 exosomal proteins exclusively expressed in the airspace of *Scnn1b*-Tg+ mice were used for this comparative analysis. Our analyses revealed that ∼46% (31 out of 67) and ∼26% (94 out of 362) of the upregulated transcripts (FC > 2 and adjusted *p*-value < 0.05) from whole lung and alveolar macrophages of *Scnn1b*-Tg+ mice, respectively, were identified as protein signatures in the BALF exosomes of *Scnn1b*-Tg+ mice (Table 3). Among these, 77% (24 out of 31) and 61% (57 out of 94) of the whole lung- and alveolar macrophage-relevant proteins, respectively, were significantly enriched in BALF exosome of *Scnn1b*-Tg+ versus WT mice. Additionally, 9 out of the 24 and 18 out of the 57 of the whole lung- and alveolar macrophage-relevant proteins, respectively, trended higher in BALF exosome of *Scnn1b*-Tg+ versus WT mice (Table 3). For example, pendrin, an anion exchanger associated with the airway surface liquid homeostasis, and its mRNA, i.e., *Slc26a4*, were significantly abundant in BALF exosomes and overexpressed in whole lung, respectively, from the *Scnn1b*-Tg+ versus WT mice. Similarly, proteins including ITGAM (CD11B), CD300A, S100A8/9, and their respective mRNAs were significantly elevated in BALF exosomes from the *Scnn1b*-Tg+ versus WT mice. The enrichment status of 13 proteins, i.e., CD177, GP2, LIPA, PIGR, SLC26A4, CD68, CTSD, RETNLA, FBP1, GPNMB, CCL9, GLA, MMP12, was consistent with their upregulated gene expression in the whole lung (Figure 7A; Table 3). The enrichment of 25 proteins, including S100A9, S100A8, ITGAM, IFITM3, SCGB3A1, SCGB3A2, BPIFB1, ADAM8, SCGB1A1, BASP1, VNN3, MFGE8, ABCG2, MLPH, CEACAM1, CYFIP2, RETNLA, FBP1, GPNMB, CD300A, STEAP3, PHLDA3, CCL9, GLA, MMP12, mirrored their upregulated gene expression in alveolar macrophages from *Scnn1b*-Tg+ versus WT adult mice. (Figure 7B; Table 3). These analyses highlight the impact of the cell/compartment-specific gene expression changes on the protein composition of the BALF exosomes.

**TABLE 3:** Comparative analysis of enriched protein signatures from *Scnn1b*-Tg+ mice (current study) and transcriptome from whole lung and purified BALF macrophage (Saini et al., 2014).

Gene signatures upregulated in whole lung or BALF macrophages or in both compartments (FC > 2; adjusted *p*-value < 0.05)	Exosomal proteins corresponding to the upregulated gene signatures in exosomes from *Scnn1b*-Tg+ versus WT mice	Enriched exosomal proteins (*Scnn1b*-Tg+ versus WT mice) (Log2FC > 1; adjusted *p*-value < 0.05)
Whole lung (67)	Out of 67 proteins corresponding to the upregulated gene signatures, a total of 31 protein signatures were present in *Scnn1b*-Tg+ BALF exosomes; 24/31 protein signatures had mean value higher in *Scnn1b*-Tg+ versus WT BALF exosomes: 24 abundant protein signatures include 9 signatures that were significantly enriched in *Scnn1b*-Tg+ versus WT BALF exosome; 4/31 protein signatures were exclusively present in *Scnn1b*-Tg+ BALF exosomes.	CD177, GP2, LIPA, PIGR, SLC26A4, CD68, CTSD, RETNLA, FBP1 Exclusively found in *Scnn1b*-Tg+ mice: GPNMB, CCL9, GLA, MMP12
BALF macrophage (362)	Out of 362 proteins corresponding to the upregulated gene signatures, a total of 94 protein signatures were present in *Scnn1b*-Tg+ BALF exosomes; 57/94 protein signatures had mean value higher in *Scnn1b-*Tg+ versus WT BALF exosomes: 57 abundant protein signatures include 18 signatures that were significantly enriched in *Scnn1b*-Tg+ versus WT BALF exosomes; 7/94 protein signatures were exclusively present in *Scnn1b*-Tg+ BALF exosomes.	S100A9, S100A8, ITGAM, IFITM3, SCGB3A1, SCGB3A2, BPIFB1, ADAM8, SCGB1A1, BASP1, VNN3, MFGE8, ABCG2, MLPH, CEACAM1, CYFIP2, RETNLA, FBP1 Exclusively found in *Scnn1b*-Tg+ mice: GPNMB, CD300A, STEAP3, PHLDA3, CCL9, GLA, MMP12
Saini et al., 2014		

## 4 Discussion

Exosome-mediated intercellular communications play important roles in normal and pathological conditions through the transfer of various bioactive compounds including proteins, lipids, DNA, and RNA molecules (Kalluri and LeBleu, 2020; De Toro et al., 2015; Stoorvogel et al., 2002). Our recent proteomic-oriented report revealed interesting findings from airspaces-derived exosomes from ozone-stressed airspaces in mice (Choudhary et al., 2021b). In this study, to gain insights into the proteomic differences and their association with the pathological endpoints, we hypothesized that alterations in the composition of exosome-bound protein signatures in *Scnn1b*-Tg+ mice are suggestive of mucoinflammatory lung disease. To investigate this, we addressed a series of questions: (1) Do *Scnn1b*-Tg+ mice exhibit differential composition of proteins in the airspace exosomes as compared to WT mice? (2) What proteins are enriched in exosomes derived from *Scnn1b*-Tg+ airspaces? (3) Do the exosomal proteins reflect macrophage activation patterns and mucoinflammatory disturbances in the *Scnn1b*-Tg+ airspace? (4) Which biological pathways are influenced by exosome-bound proteins in *Scnn1b*-Tg+ versus WT mice?

Protein signatures found enriched in the BALF exosomes from WT mice were suggestive of normal tissue functions. For example, homeodomain-only protein homeobox (HOPX), which is required for developing mouse neocortex (Vaid et al., 2018), was enriched in WT mice. Glutathione peroxidase 8 (GPX8), which protects cytosol from H_2_O_2_ leakage in the endoplasmic reticulum (Ramming et al., 2014), and the growth factor midkine (MDK) which regulates the renin-angiotensin system and mediates the interaction between the kidneys and lungs in mice (Hobo et al., 2009), were enriched in WT mice BALF exosomes. Interestingly, several exosomal proteins that were exclusively expressed in WT mice BALF exosomes were also involved in normal biosynthesis, i.e., keratin, type I cuticular Ha2 (KRT32), a type I hair keratin, involved in exoskeleton formation (Adav et al., 2023), pleckstrin homology domain-containing, family G member 5 (PLEKHG5), a guanine-nucleotide exchange factors to activate ras homolog family member A (RhoA) (De Toledo et al., 2001; Qian et al., 2019), and antigen peptide transporter 1 (TAP1), the subunit of transporter associated with antigen processing (Gaudet and Wiley, 2001a; Gaudet and Wiley, 2001b). These data indicate that under homeostatic conditions, exosome-bound proteins in bronchoalveolar airspaces are relevant to normal physiological processes.

A large number of protein signatures relevant to inflammation, macrophage activation, and mucous cell metaplasia were found enriched in the BALF exosomes from *Scnn1b*-Tg+ mice. CD177, a specific marker of neutrophil activation (Lévy et al., 2021; Bayat et al., 2010), was the most abundant protein present within the exosomes from *Scnn1b*-Tg+ mice. Extracellular vesicle-derived ITGAM, which is involved in acute lung injury (Hu et al., 2023), was highly expressed in the exosome of *Scnn1b*-Tg+ versus WT mice. Neutrophil elastase (NE), a key inflammatory protease (Voynow and Shinbashi, 2021; Pham, 2006), and a known promoter of mucous cell metaplasia in the *Scnn1b*-Tg+ mice (Gehrig et al., 2014), Lymphocyte antigen 6 family member G (Ly6G) and MPO, two distinct neutrophil markers (Lee et al., 2013; Aratani, 2018), and CTSG, a serine protease, acting as a chemoattractant for mononuclear cells and neutrophils (Chertov et al., 1997), were exclusively expressed in BALF exosomes of *Scnn1b*-Tg+ mice. Moreover, MMP-9 and MMP-12, the proteolytic enzymes, were also exclusively expressed in the BALF exosome of *Scnn1b*-Tg+ mice. Of note, the roles of MMP-9 and MMP-12 are well-established in the pathogenesis of COPD (Molet et al., 2005; Papakonstantinou et al., 2015; Liu et al., 2020).

Our previous report indicated that an admixture of activated macrophages was present in the BALF of *Scnn1b*-Tg+ mice, with predominantly classically-activated (M1) macrophages in neonates and alternatively-activated (M2) macrophages in adulthood (Saini et al., 2014). Consistent with this report, M2 activation-relevant protein signatures showed a significant enrichment within the BALF exosomes of *Scnn1b*-Tg+ mice. CHIL3/YM1, an M2 alternative activation marker in mice (Raes et al., 2005; Raes et al., 2002), forms eosinophilic crystals in the cytoplasm of alveolar macrophages and multinucleate giant cells, and within the alveolar spaces (Guo et al., 2000). YM1/2 contributes to Th2 cytokine production and allergic airway inflammation (Cai et al., 2009). *Chil3*, an mRNA for CHIL3/YM1, showed significantly upregulated expression in the alveolar macrophage of *Scnn1b*-Tg+ mice (Saini et al., 2014). Consistent with this report, CHIL3/YM1 was enriched in the BALF exosomes from *Scnn1b*-Tg+ mice and the macrophage-specific immunolocalization of this protein suggests its origination from macrophages. Galectin-3 (LGALS3), a β-galactoside-binding lectin, is also expressed by alternatively activated macrophages (MacKinnon et al., 2008; Nishi et al., 2007). Galectin-3 was enriched in BALF exosomes from ozone-exposed mice (Choudhary et al., 2021b). Galectin-3 was exclusively enriched in airspace-derived exosomes from *Scnn1b*-Tg+ mice and had macrophage-specific immunolocalization in *Scnn1b*-Tg+ lungs. FIZZ1 (Found in Inflammatory Zone 1/RETNLA), another well-known alternative macrophage activation marker in mice (Raes et al., 2002), was found significantly upregulated in the alveolar macrophages of *Scnn1b*-Tg+ lungs (Saini et al., 2014). The airway epithelial-specific immunolocalization of FIZZ1 has been reported in hypoxia-exposed (Teng et al., 2003) and ozone-exposed mice (Choudhary et al., 2021b). Consistent with these reports, FIZZ1 immunostaining was localized to the airway epithelium of *Scnn1b*-Tg+ mice. These reports suggest that exosome-bound FIZZ1 originates from airway epithelial cells in *Scnn1b*-Tg+ airspace. While we were able to identify the cellular sources of exosomes-bound protein signatures, it remains unclear whether the exosomes continue to exchange their cargo after their extracellular release.

Mucoinflammatory lung diseases are characterized by intrapulmonary accumulations of hyper-concentrated mucus, which contributes to the vulnerability to recurrent infections (Boucher, 2019; Livraghi-Butrico et al., 2017). To investigate the BALF exosome-bound protein signatures potentially relevant to the development of lung mucoinflammation, we compared the presence of protein signatures associated with mucoinflammatory lung diseases in the BALF exosomes of *Scnn1b*-Tg+ mice versus WT mice. Numerous proteins previously shown to be involved in mucoinflammatory lung diseases were enriched in the exosomes from *Scnn1b*-Tg+ mice, including S100A9, CAMP, S100A8, PGLYRP1, LTF, BPIFB1, LIPA, APOB, APOC3, CD5L, CD14, CTSD, and LGALS3. *Scnn1b*-Tg+ mice present with neutrophilic inflammation in airspace (Mall et al., 2004) and enriched S100A8 was found in airspaces-derived exosomes of ozone-challenged mice (Choudhary et al., 2021b). These findings were consistent with the increased S100A8 expression in BALF exosome and neutrophils of *Scnn1b*-Tg+ mice (Figure 3C; Figures 4A, B). Similarly, the upregulation of protein signatures relevant to MCM, i.e., SCGB1A1 and CLCA1, was consistent with the previous study that *Scnn1b*-Tg+ mice have increased numbers of mucous cells in airways versus their WT littermates (Mall et al., 2008).

BALF exosome-bound proteins from stressed airways might contribute to the functional and disease-associated pathways (Choudhary et al., 2021b). Ingenuity pathways analysis (IPA) on the differentially expressed proteins within the exosomes gathered in this study revealed activation of several canonical pathways including neutrophil degranulation, antimicrobial peptides, regulation of TLR by endogenous ligand, neutrophil extracellular trap signaling pathway, and Fc-gamma receptor-dependent phagocytosis. Additionally, enriched protein-protein interaction (PPI) networks from airspace-derived exosomes from BALF of *Scnn1b*-Tg+ mice revealed the major subsets including neutrophil degranulation, immune system, innate immune system, and antimicrobial peptides. However, the PPI analyses from protein signatures upregulated and exclusively expressed in WT mice presented endoplasmic reticulum-associated networks including the endomembrane system, endoplasmic reticulum, and endoplasmic reticulum membrane, which might potentially help maintain the homeostatic conditions of biosynthesis.

Increasing research on exosomes have showed its potential in translational medicine and have provided novel insights for the clinical diagnostics and therapeutic strategies as biomarkers, vaccines, and drug carriers (Khan et al., 2015; Alvarez-Erviti et al., 2011; Tan et al., 2014; Li et al., 2013). This is the first study to provide a detailed proteomics analysis of exosomes from *Scnn1b*-Tg+ airspaces and our findings revealed that BALF exosomes-bound protein signatures reflect the muco-obstructive lung disease-relevant disturbances. These protein signatures could be used for diagnostic or prognostic monitoring of patients with muco-obstructive lung disease, as well as to design novel exosome-based therapies. There are also limitations to this study. First, this study characterized proteomic signatures in male mice only. Although the size and concentration of the BALF exosome in *Scnn1b*-Tg+ male and *Scnn1b*-Tg+ female animals were comparable, the sex-specific differential enrichment of exosomal proteins cannot be ruled out. Second, we were unable to perform functional studies to determine the biological influence of exosome-derived proteins in cell-based or animal models.

Collectively, our data provides a detailed proteomics analysis of the exosomes from *Scnn1b*-Tg+ and WT mice airspaces. With the immunolocalization approach, we were able to identify cellular sources of some of the disease-relevant proteins. The comparative analysis between enriched BALF exosome proteins and upregulated gene transcriptomes in *Scnn1b*-Tg+ versus WT mice revealed the relevance of the mRNA level changes and their impact on the protein signatures in the airspaces. We believe this study will create a foundation for future mechanistic studies aiming at understanding the pathogenesis of mucoinflammatory lung diseases and facilitating the identification of novel molecular and cellular processes for the eventual therapeutic targeting during the progression of pulmonary diseases. This study also suggests that the exosomes from BALF carry disease-relevant protein signatures that can be used as diagnostic predictive biomarkers for mucoinflammatory diseases.

## Data Availability

The original contributions presented in the study are included in the article/Supplementary Material. Our raw data is available in MassIVE database (https://massive.ucsd.edu/ProteoSAFe/dataset.jsp?accession=MSV000095568). Further inquiries can be directed to the corresponding author.

## Supplementary material

SUPPLEMENTARY FIGURE S1 Concentration **(A)** and size **(B)** of isolated exosome. Each exosome sample represents exosomes collected from BALF harvested from a total of one, two, or three adult (6–7 weeks old) mice. Each mouse has the same volume in all the exosome samples. Error bars represent Mean ± Standard Error of the Mean (SEM).

SUPPLEMENTARY FIGURE S2 Low resolution of immunohistochemical staining for panel B and C of Figure 3. The broad area of the lung section images for immunohistochemical staining of RETNLA/FIZZ1 **(A)**, CHIL3/YM1 **(B)**, LGALS3/Galectin-3 **(C)**, and S100A8/MRP8 **(D)** were captured at 4× objective.

SUPPLEMENTARY FIGURE S3 High resolution heat maps for panel A of Figure 4 depicting normalized protein abundance values (Z-scores) of exosome-bound proteins associated with mucoinflammatory disease (mice and humans) and mucous cell metaplasia. The white blanks indicate the levels below the limit of detection.
