# Supplementary figures and images for: Airspaces-derived exosomes contain disease-relevant protein signatures in a mouse model of cystic fibrosis (CF)-like mucoinflammatory lung disease
Source: Front Pharmacol. 2024 Sep 25;15:1460692. doi: 10.3389/fphar.2024.1460692 (PMC11461968; doi:10.3389/fphar.2024.1460692)

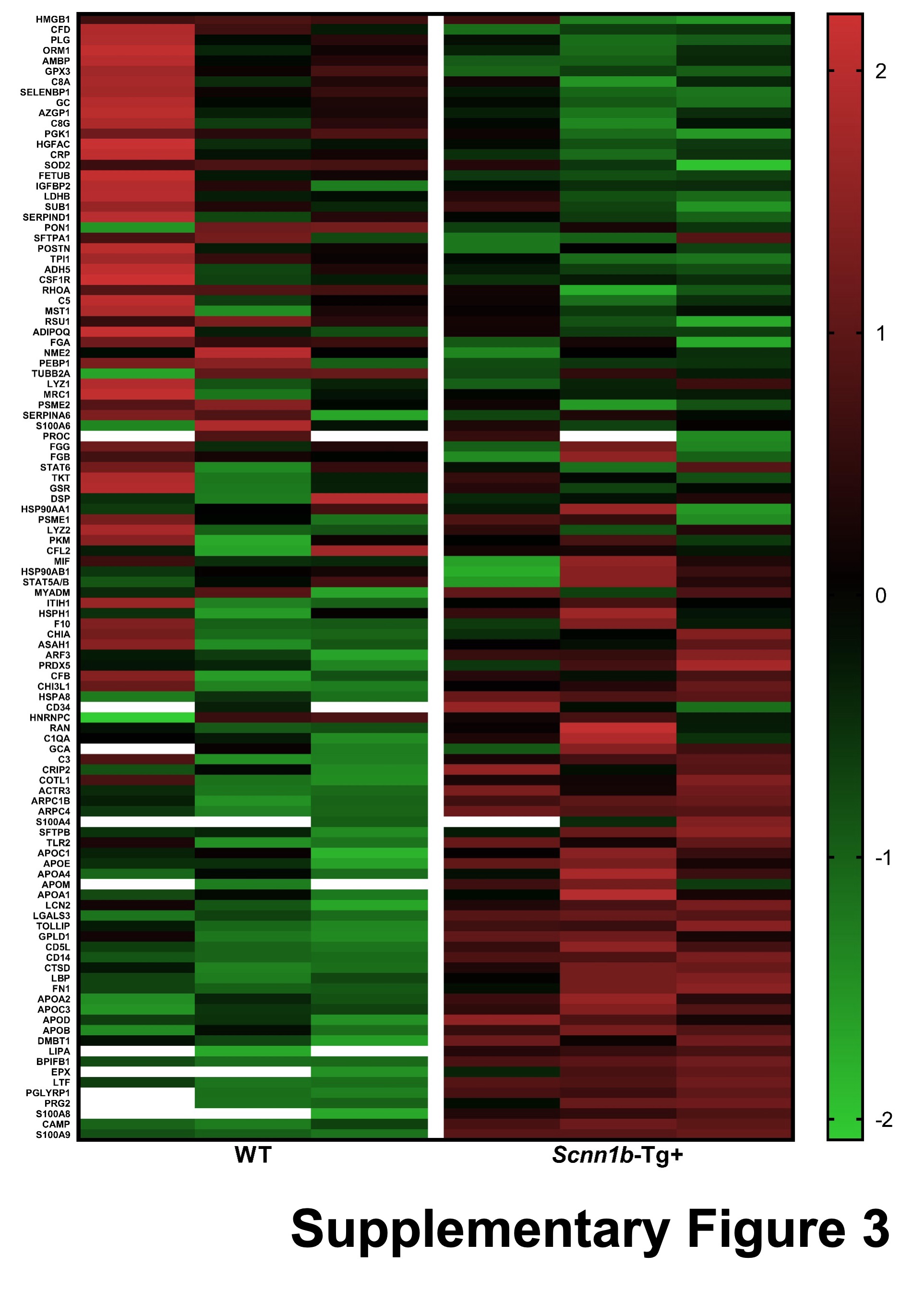

Supplement: Supplementary file 1 [file Image3.jpeg]

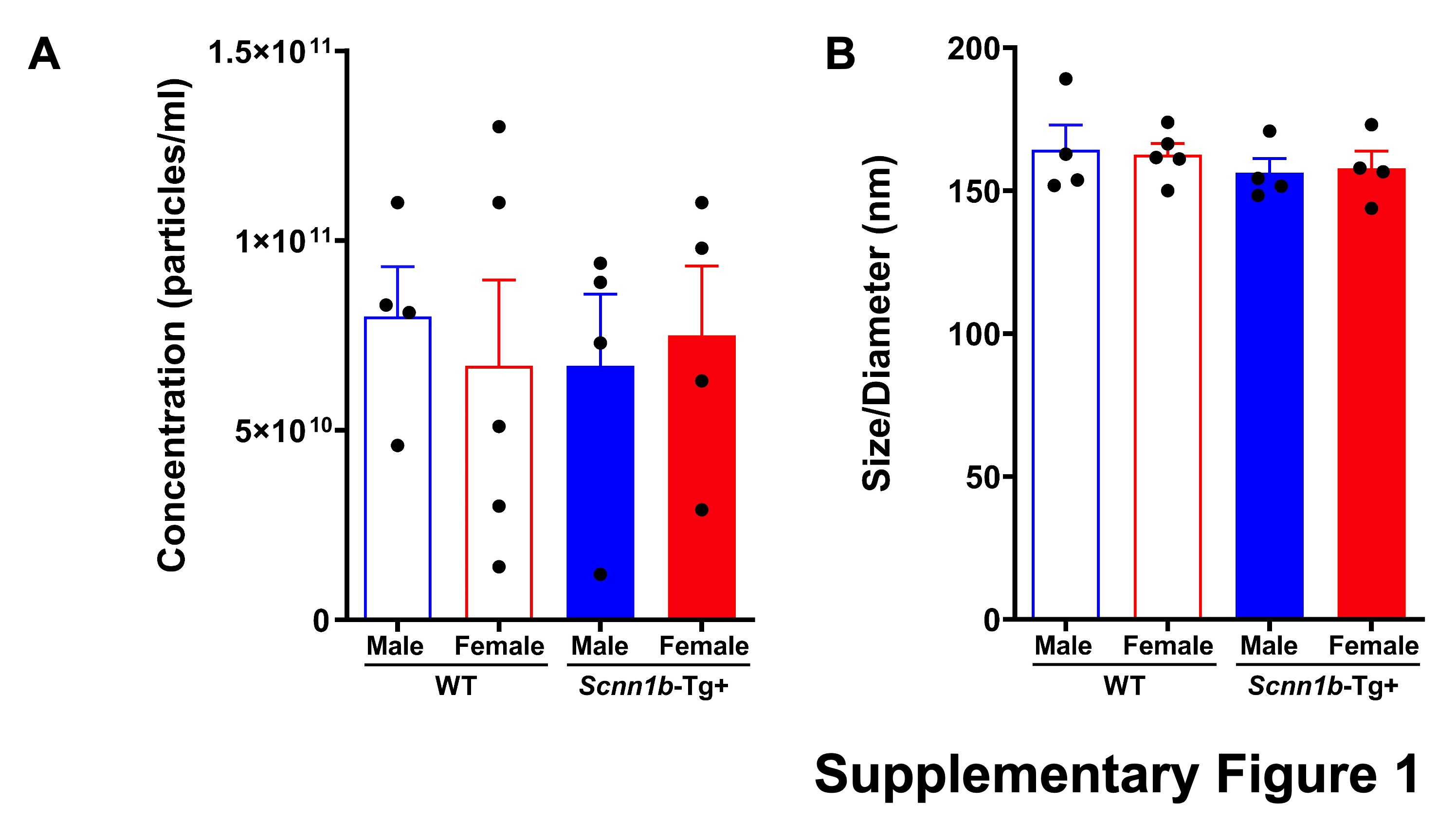

Supplement: Supplementary file 2 [file Image1.jpeg]

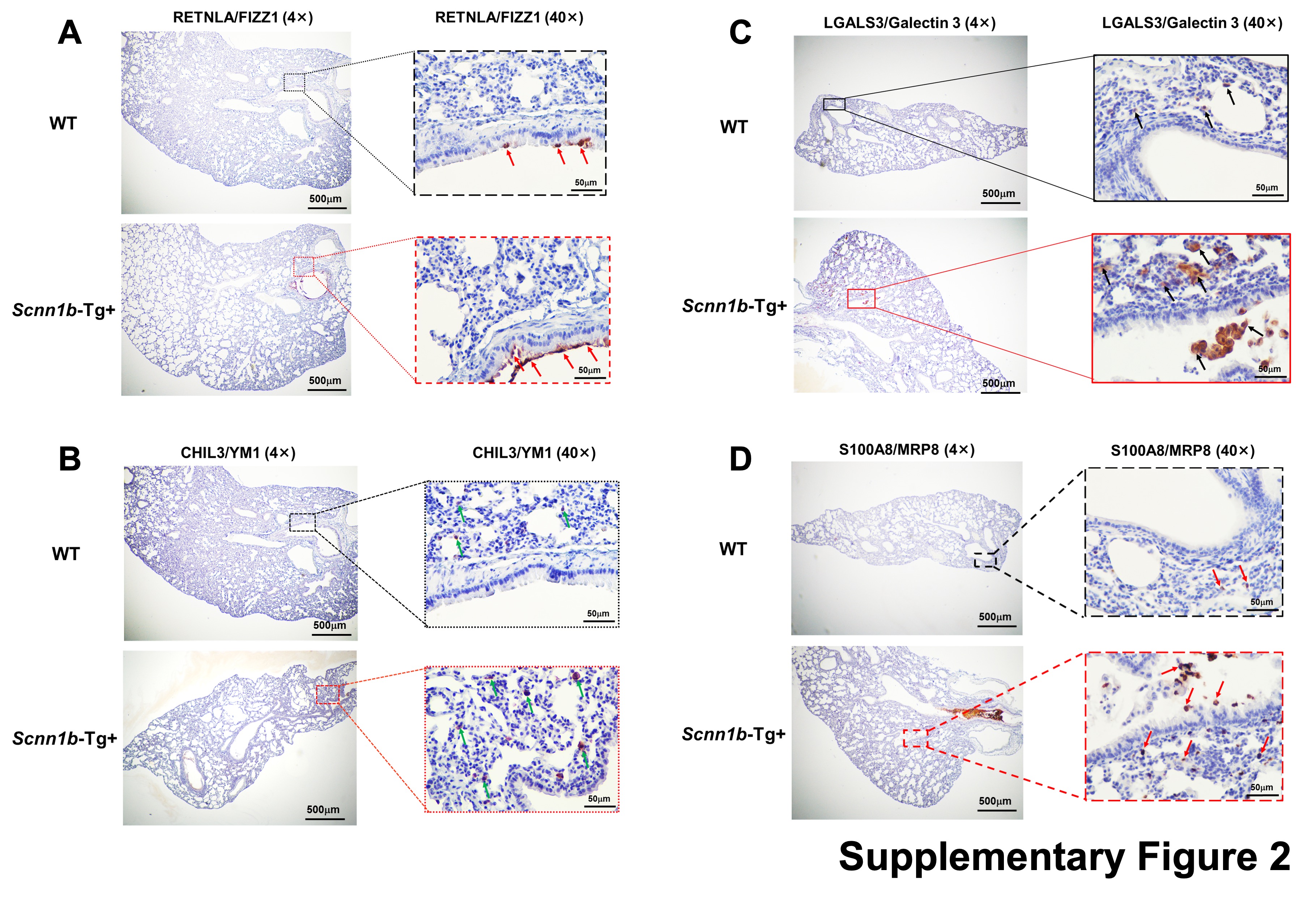

Supplement: Supplementary file 3 [file Image2.jpeg]
